# Active Targeted Nanoformulations via Folate Receptors: State of the Art and Future Perspectives

**DOI:** 10.3390/pharmaceutics14010014

**Published:** 2021-12-22

**Authors:** Cristina Martín-Sabroso, Ana Isabel Torres-Suárez, Mario Alonso-González, Ana Fernández-Carballido, Ana Isabel Fraguas-Sánchez

**Affiliations:** 1 Department of Pharmaceutics and Food Technology, School of Pharmacy, Complutense University, 28040 Madrid, Spain; crmartin@ucm.es (C.M.-S.); galaaaa@ucm.es (A.I.T.-S.); marioalonsogonzalez@ucm.es (M.A.-G.); afernand@ucm.es (A.F.-C.); 2 Institute of Industrial Pharmacy, Complutense University, 28040 Madrid, Spain

**Keywords:** etarfolide, folic acid, mirvetuximab soravtansine, ovarian cancer, rheumatoid arthritis, targeted therapies, theranostic, vintafolide

## Abstract

In normal tissues, the expression of folate receptors is low and limited to cells that are important for embryonic development or for folate reabsorption. However, in several pathological conditions some cells, such as cancer cells and activated macrophages, overexpress folate receptors (FRs). This overexpression makes them a potential therapeutic target in the treatment of cancer and inflammatory diseases to obtain a selective delivery of drugs at altered cells level, and thus to improve the therapeutic efficacy and decrease the systemic toxicity of the pharmacological treatments. Two strategies have been used to achieve this folate receptor targeting: (i) the use of ligands with high affinity to FRs (e.g., folic acid or anti-FRs monoclonal antibodies) linked to the therapeutic agents or (ii) the use of nanocarriers whose surface is decorated with these ligands and in which the drug is encapsulated. This manuscript analyzes the use of FRs as a target to develop new therapeutic tools in the treatment of cancer and inflammatory diseases with an emphasis on the nanoformulations that have been developed for both therapeutic and imaging purposes.

## 1. Introduction

**Figure 1:**
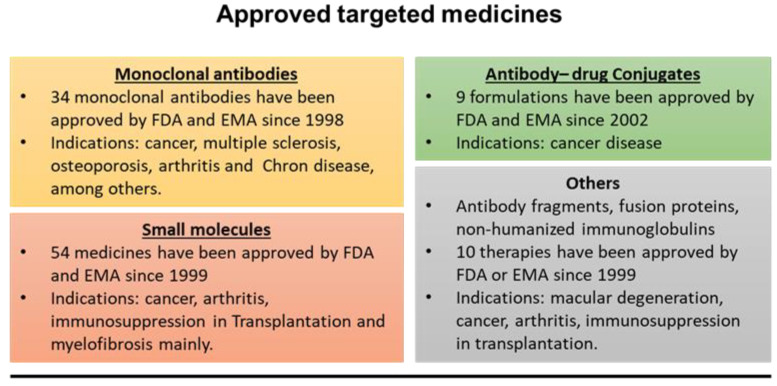
Summary of the type of targeted medicines that have been approved by FDA and/or EMA.

In recent decades, the use of targeted therapies that act selectively on target cells has increased considerably [1,2]. To obtain this selective location of the drugs at the therapeutic targets, several strategies can be used. One of the most attractive and evaluated approaches is the conjugation of the therapeutic agent to a targeting moiety (developing nanoconjugates) or to the surface of a nanocarrier used for its vehiculization, which is specifically recognized by the pathologic cell, tissue, or organ [3,4,5]. In this way, a great number of ligands have been used, including antibodies, peptides, aptamers, carbohydrates, or vitamins [6,7,8,9]. In fact, 107 targeted medications have already been approved by the US Food and Drug Administration (FDA) and/or European Medicines Agency (EMA) for the treatment of different disorders (Figure 1).

**Figure 2:**
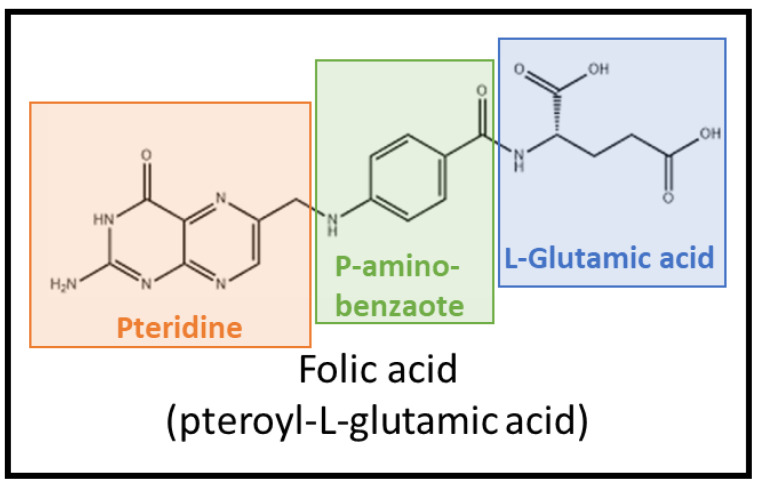
Structure of folic acid.

**Figure 3:**
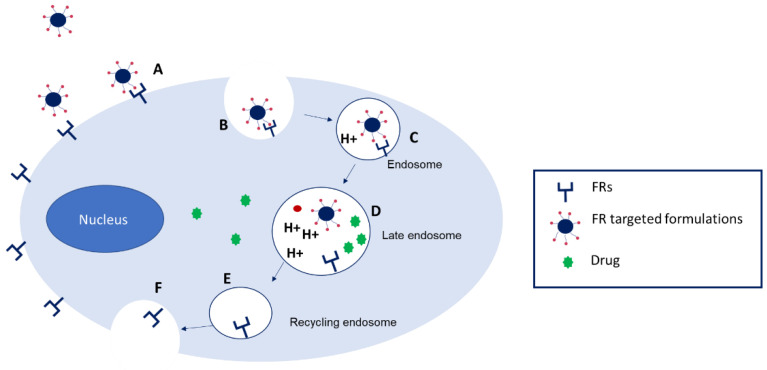
Scheme showing folic acid- and FR-targeted formulations internalization. Folic acid or another ligand binds to folate receptor (**A**). An invagination of the plasma membrane is generated (**B**) and the folate receptor-ligand conjugate is enclosed in a vesicle named endosome (**C**), where the acidic pH triggers conformational changes in the folate receptors, inducing the release of the ligand (**D**) and the drug into the cells, and the recycling of the folate receptor to the cell surface (**E**,**F**).

Folic acid (pteroyl-l-glutamic acid) is a widely used targeting ligand, especially in cancer therapy (Figure 2). It is the synthetic form of folate (pteroyl-l-glutamate), also known as vitamin B_9._ It should be mentioned that both terms, folic acid and folate are often used interchangeably but folic acid does not occur in nature [10]. This is a crucial molecule indispensable for DNA replication and repair, RNA synthesis, as it participates in nucleotide synthesis, amino acid metabolism, and phospholipid biosynthesis [11,12,13]. At the plasma membrane, folate is transported mainly through three different mechanisms: proton-coupled folate transporter (PCFT, also known as SLC46A1), reduced-folate carrier (RFC-1, also known as SLC19A1), and folate receptor (FR). PCFT that catalyzes symport of protons with the folate substrate is most active at acidic pHs, and is responsible for the intestinal absorption of this vitamin [11]. RFC-1 is a ubiquitously expressed antiporter in the intestine, hepatocytes, choroid plexus, and renal epithelial cells that uses the gradient of organic phosphate across the cell membrane to allow folate uptake at neutral pH and to show a low folate-binding affinity (K_m_ = 1–10 μM) [14,15]. By contrast, FRs are high affinity folate-binding cysteine-rich glycoproteins that mediate folate uptake via receptor-dependent endocytosis (Figure 3). The binding of FR ligands is followed by the invagination of the plasma membrane around the receptor-ligand conjugate, forming an endosome. The acidification of the endosomal environment through the action of proton pumps induces a conformational change in FR that triggers the release of the ligand into the cell. Then, FR is recycled again to the cell surface [16,17]. 

In human, four isoforms of FRs have been identified: FRα, FRβ, FRγ, and FRδ. FRα, FRβ and FRδ are glycosyl-phosphatidylinositol-anchored membrane proteins. However, FRγ is primarily a secretory protein [18]. In healthy tissue, FR expression is largely limited to cells important for embryonic development, such as neural tube or placenta, and for folate reabsorption, being also expressed in kidneys. In this way, in general, in normal tissues their expression is low. However, in certain circumstances such as activated macrophages or in cancer cells, they are overexpressed, becoming a good target to get selective therapy in situations such as cancer or inflammatory diseases [12,19,20]. It should be denoted that while cancer cells overexpress FRα, in activated macrophages, FRβ are overexpressed. Moreover, FRα are also expressed at the choroid plexus epithelium and may represent a potential for targeting the central nervous system [21].

This manuscript analyses the use of folate receptors as a target to improve the treatments of both cancer and inflammatory diseases, pathologies where they have shown important potential, by describing the nanoformulations that have been developed for both diagnostic and therapeutic purposes. 

## 2. Folic Acid-Targeted Chemotherapy

As aforementioned, while FRs are overexpressed in many tumors, such as ovarian, lung, colon, and endometrial carcinomas among others, their expression in normal cells is limited, making them, especially FRα that are the most common overexpressed subtype, interesting tumor-associated antigens in cancer therapies [22,23]. Interestingly, it has been reported that ovarian cancer, specifically epithelial ovarian cancer subtype, not only overexpresses FRα (found in more than 80% of the cases), but also that this overexpression is related to tumor grade and disease outcome [24], suggesting that FRs participate in ovarian cancer progression and that they are a good strategy to develop targeted therapies. In fact, ovarian cancer is probably the neoplasm where the use of FR for targeted therapies have been exploited the most, with several formulations under clinical research. On the contrary, in colon cancer the frequency of FRα positive tumors is low, with these receptors being overexpressed in around 30–40% of human colorectal carcinoma tissues [25].

**Figure 4:**
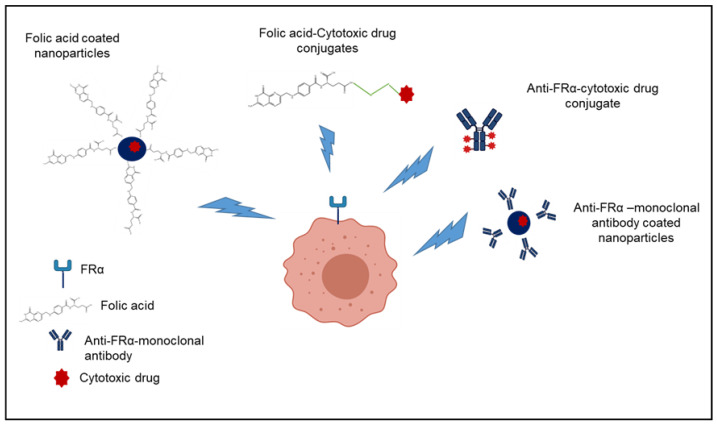
Summary of FR-targeted nanoformulations developed for cancer therapy.

To target FRs in cancer therapies, two strategies have been evaluated: the use of folic acid or other chemical entities with a high affinity for FRs and the use of anti-FRs-monoclonal antibodies [26]. In both cases, the chemotherapeutic agent is directly conjugated to FR ligands or encapsulated into a nanocarrier whose surface has been “decorated” with the targeting moiety with the aim of obtaining a selective delivery of the antineoplastics at tumor level (Figure 4), enhancing the anticancer activity and decreasing chemotherapy-related toxicity. In this way, a huge number of nanoformulations have been developed and evaluated to obtain a selective chemotherapeutic effect. Folate-drug conjugates and folate-bounded radiopharmaceuticals have also been developed. Table 1 displays the developed FR-targeted nanoformulations for precision medicine in cancer disease (Table 1).

### 2.1. Folic Acid Conjugated Nanomedicines 

#### 2.1.1. Folic Acid-Cytotoxic Drug Conjugates 

Ladino and co-workers reported for the first time in 1997, the utility of folate-cytotoxic agent conjugates to improve cancer chemotherapy. These researchers developed folate-maytansinoid (a potent microtubule-inhibiting compound) conjugates and demonstrated that they entered to cells exclusively via FR-mediated endocytosis, showing a marked antiproliferative effect in FR-positive cells, including ovarian, colon, and cervical cancer cells; and a non-activity on FR-negative melanoma and breast cancer cells [27]. These results were also confirmed in lung tumor models developed in mice. While in M109-derived lung tumors (M109 cells overexpress FR) a significant tumor growth reduction in around 30% was achieved, which reverted by the previous administration of free folic acid with the aim of saturating FRs and avoiding the binding of FR-olate-maytansinoid conjugate, in A549-derived tumors (A549 are FR-negative lung cancer cells) a significant tumor growth inhibition was not detected [28]. All these results indicate that the development of folic acid-antineoplastic conjugates is a good strategy to obtain selective anticancer therapies. Since them, numerous folate-antineoplastic conjugates have been developed and evaluated in several FR-positive tumors [29]. For example, 5-fluorouracil derivates conjugated with folic acid demonstrated to exert a potent anticancer activity in colon cancer models, even in tumors that were resistant to 5-fluorouracil [30]. While non-conjugated drugs showed an IC_50_ value of 4.30 nM, the IC_50_ value of folic acid conjugates was significantly lower (0.180 × 10^−10^ nM). Similar results were also found in folic acid-methotrexate-arabinogalactan conjugates that showed a 6.5-fold increased anticancer effect in tumors overexpressing FRs [31], or in folate-SB-T-1214 conjugates (SB-T-1214 is a potent taxoid). Interestingly, this last formulation demonstrated a significantly higher cytotoxicity in FR-positive cancer cells than in normal cells [32]. Folic acid-mitomycin conjugates were also effective in lung adenocarcinoma cells that overexpress FRα. In vitro studies demonstrated that they were efficiently internalized by these cells exhibiting a potent antiproliferative activity and that this activity was attributed to an enhanced internalization via folate receptor endocytosis as an excess of folic acid in the medium blocked this cytotoxic effect. FR-negative cervix cancer cells were unresponsive. In vivo studies in mice also confirmed these results, suggesting that the conjugation to folic acid is a good strategy to obtain selective anticancer activity of mitomycin C [33]. Folic acid-bleomycin conjugates were also effective as a cytotoxic formulation in FRα overexpressing cells [34]. 

The conjugation with folic acid is especially interesting in drugs that show a severe toxicity that limits their clinical use. This is the case in camptothecin, which shows a potent anticancer efficacy in several tumor types like ovarian neoplasms, but its clinical use is very limited due to its severe toxic effects such as hemorrhagic cystitis and myelotoxicity [35,36]. Folic acid-camptothecin conjugates using polyethylene glycol (PEG) as linker, demonstrated a selective FR-mediated cytotoxicity [37]. Studies carried out in human KB cells (subline of the Keratin-forming tumor cell line HeLa) showed that while in the absence of excess of free folic acid, these camptothecin conjugates had an IC_50_ of 6 nM after 72 h of incubation; in the presence of an excess of folic acid the IC_50_ was significantly higher with a value of 0.1 mM. Although further studies are needed, these results indicate that folic acid would allow a selective delivery of camptothecin at the tumor level, which would decrease its related toxicity. This selective anticancer effect was also detected in folic acid-polyethylene glycol-rhaponticin conjugate that demonstrated a selective cytotoxicity in FR-positive cells. While this conjugate showed a marked antiproliferative effect in KB cells (FR-positive cells), in 4-T1 cells (FR-negative breast cancer cells), the conjugate was ineffective. A significant tumor growth reduction was also detected in tumor models developed in mice expressing FR (derived from KB and M109 cells). Interestingly, a reduced in vivo toxicity was detected in conjugated drugs [38], reinforcing the development of folic acid conjugates for a selective chemotherapy. In fact, a folic acid conjugate known as vintafolide, which consists of folic acid conjugated to a vinca alkaloid (vinblastine) by a peptide linker, has been reported to be useful in the treatment of ovarian cancer and has reached clinic [39]. Other two folic acid conjugates BMS-753493, EC1456 have also reached the clinical stage. Both of them are explained in detail in Section 6. 

However, the conjugation of folic acid to other commonly used antineoplastics such as paclitaxel or cisplatin were not so effective, and a higher efficacy of conjugated drugs was not observed compared with non-conjugated antineoplastics in FR-positive tumors. While non-conjugated paclitaxel, in a drug concentration of 200 nM, produced a decrease on cell proliferation higher than 50% on FR-positive cells (KB and M109), the antiproliferative effect of folic acid-conjugated paclitaxel was lower than 30% when administered at the same concentration [40]. Similar results were also found in folic acid-polyethylene glycol-cisplatin conjugates. In this case, a higher uptake was detected in lung cancer cells overexpressing FRs. However, this increased internalization did not result in a higher antiproliferative effect. The authors explained that the conjugates were neutralized or blocked during the folate receptor-mediated endocytosis process and were not able to effectively reach the nuclear DNA [41]. The lack of an improved efficacy could be related to the chemical modification of the drug required for its conjugation.

#### 2.1.2. Folic Acid-Functionalized Nanoparticles

Apart from folate-cytotoxic conjugates, another strategy to obtain selective delivery of the chemotherapeutic agents in tumors that overexpress FRs is the use of folic acid-coated nanocarriers for their vehiculization. In this way, numerous nanomedicines, based on polymeric, lipidic, or metallic carriers, have been developed [42,43,44]. Folic acid-coated liposomes loaded with doxorubicin or paclitaxel have been successfully developed, showing a higher uptake than uncoated formulations and higher anticancer activity in FR-positive breast tumors. For example, folic acid-coated liposomes loaded with doxorubicin showed a significantly higher tumor growth inhibition (with values around 68%) in triple-negative breast cancer models than uncoated liposomes (with a tumor growth inhibition around 56%) [45]. Similar results were also found in folic acid-coated liposomes loaded with paclitaxel in triple-negative breast tumor models, showing in all cases that the incorporation of folic acid significantly increased the anticancer activity against this neoplasm [45,46,47]. In fact, both paclitaxel- or doxorubicin-loaded folate receptor-targeted liposomes were even effective in decreasing in vivo the growth of breast cancer metastases to bone or to lung, showing a higher accumulation metastatic lesion than in normal bone or healthy lungs [48,49]. 

Folic acid-coated silica mesoporous nanoparticles have also been developed to obtain selective delivery of several antineoplastics. For example, the anticancer effect of doxorubicin in HeLa cervical cancer cells was enhanced using folic acid-coated silica nanoparticles as nanocarriers. While non-coated silica nanoparticles exhibited a tumor growth inhibition around 78%, coated nanoparticles showed a significantly higher inhibition rate around 90% [50]. Silica nanoparticles coated with folic acid and loaded with cisplatin have also been designed, showing a higher uptake and an increased cytotoxicity than uncoated counterparts in these FR-overexpressing cervical cancer cells. However, their effect was not tested in vivo [51]. Finally, folic acid-coated silica nanoparticles significantly increased the antiproliferative effect of topotecan, in vitro in Y79 retina cancer cells overexpressing FR receptors and in vivo in tumors derived from these cells and subcutaneously formed in mice. While free topotecan exhibited a tumor growth inhibition of around 26%, non-coated and folic acid-coated nanoparticles showed a higher tumor reduction of around 47% and 75%, respectively [52]. 

Numerous polymer-based nanocarriers have also been studied for FR targeting [42]. Poly(lactic-co-glycolic acid) is a common polymer in the development of drug delivery systems, and has been used to elaborate nanoparticles coated with folic acid. These formulations have demonstrated to exhibit a selective cisplatin and paclitaxel delivery in non-small cell lung cancer and bone metastatic breast cancer animal models that overexpress FRs [53,54]. However, this selective delivery did not result in an improved anticancer effect in FR-positive lung tumors (derived from M109 cancer cells), as folic acid-coated nanoparticles showed a similar tumor growth inhibition than uncoated counterparts [55]. 

Finally, numerous researchers have also coated metallic nanoparticles with the aim of obtaining their selective accumulation at the tumor mass. For example, folic acid-functionalized iron oxide condensed colloidal magnetic clusters containing doxorubicin as antineoplastic agent, showed a higher cellular uptake in FR overexpressing triple-negative breast cancer cells (MDAMB-231) than in hormone receptor positive breast cancer cells that lack of FRα expression (MCF7 cells). A higher cytotoxicity was also detected in FR-positive tumoral cells and attributed to the higher internalization [56]. This nanoformulation also demonstrated a higher internalization in FR-positive cervical cancer cells, enhancing the antiproliferative effect of radiation [57]. 

Apart from targeting cancer cells, some researchers have also exploited the opportunity to target tumor-associated macrophages (TAM). Macrophages are divided in two categories: M1 and M2. While M1 macrophages inhibit tumor progression due to the release of tumor-killing molecules such as ROS and NO and immunostimulatory cytokines such as IL-1, IL-6, and TNF that have an antitumor effect, M2 macrophages facilitate the progression of the tumors, by promoting tumor invasion and metastases, tumor angiogenesis, and tumor immunosuppression [58,59]. At tumors, macrophages express M2-like phenotype [60]. In this way, their ablation, or their re-differentiation into M1 macrophages would exert an anticancer effect. Interestingly, FRβ is overexpressed in M2-polarized-TAM, and the targeting of this receptor could be a good strategy to obtain selective delivery of the therapeutics agents in these macrophages. For example, folic acid-coated liposomes loaded with BIM-S plasmid, which induce apoptosis, have been designed with the aim of obtaining selective delivery into M2-TAM in non-small cell lung cancer, showing a tumor growth inhibition in tumors generated in mice using LL/2 cells (FR positive). While non-decorated BIM-S loaded liposomes showed a tumor growth inhibition compared with the control of around 30%, folic acid-coated BIM-S-loaded liposomes exhibited a significantly higher tumor growth inhibition (around 90%), due to the accumulation of these nanoparticles at TAM [61]. Folic acid-functionalized liposomes loaded with doxycycline, as an inhibitor of M2 polarization [62], were also effective in this cancer type [63]. Finally, folic acid-coated chitosan nanoparticles, loaded with signaling transducers and activators of transcription 3 (STAT) silencing siRNA sequence with the aim of targeting M2-TAM to induce their M1-redifferentiation, have demonstrated promising results in the Lewis lung carcinoma model. While non-coated formulation showed a tumor growth reduction of around 50%, folic acid-coated systems exhibited a growth inhibition of around 75% [64]. 

### 2.2. Anti-FR-Monoclonal Antibody-Coupled Nanoformulations 

A second targeting approach to obtain selective delivery in FRs overexpressing tumors has used anti-FR-monoclonal antibodies, and several nanoformulations have been designed and evaluated. 

#### 2.2.1. Anti-FR-Monoclonal Antibody-Drug Conjugates

The use of monoclonal antibodies against FRα is a good approach to obtain selective accumulation at the tumors, as this is the subtype that is overexpressed in cancer cells. Farletuzumab (also known as MORAb-003) is a fully-humanized monoclonal antibody that targets FRα and exerts a cytotoxic activity by inducing cell death via antibody-mediated cellular cytotoxicity and complement-dependent cytotoxicity and by promoting autophagy-associated cell death. It also inhibits the association of FRα and Lyn kinase, reducing the proliferative advantage of cancer cells [22]. Farletuzumab has even reached clinical investigation and several studies have evaluated their effect in ovarian cancer patients [65,66], demonstrating to be useful in combination with paclitaxel or carboplatin [67]. This anti-FRα-antibody has been conjugated with eribulin, a potent microtubule-targeting agent using several cleavage linkers, demonstrating a selective antitumor efficacy in non-small cell lung cancer xenograft models [68]. This antibody-drug conjugate is being evaluated in a phase I clinical trial in patients with ovarian and non-small-cell lung cancer (NCT03386942).

Monoclonal antibody mirvetuximab, which targets FRα, has also been conjugated with antineoplastic drugs, specifically with maytansinoid DM4, a potent cytotoxic agent. This antibody-drug conjugate, known as mirvetuximab soravtansine has demonstrated a high anticancer activity in vitro and in vivo in animal models of FR-overexpressing tumors such as ovarian and non-small lung carcinomas not only as monotherapy [69], but also in combination with bevacizumab, carboplatin, and doxorubicin in ovarian cancer models [70]. In fact, several clinical trials are ongoing to evaluate its anticancer effect in ovarian and endometrial cancer among others (NCT03835819, NCT0383236, NCT04296890, and NCT04606914) [71,72,73,74].

#### 2.2.2. Anti-FR-Monoclonal Antibody-Functionalized Nanoparticles

Although most of the FR-targeting nanomedicines are based on the incorporation of folic acid, some anti-FR-antibody-conjugated nanoparticles have also been developed. For example, antibody-conjugated gold-coated magnetite nanoparticles have been prepared and evaluated in FRα overexpressing triple-negative breast cancer models, showing a selective cytotoxicity due to their accumulation at tumor sites via FR mediated internalization [75]. 

**Table 1:** FR-targeted nanoformulations designed for cancer treatment.

		Formulation	Cancer type/Activity	Stage	Reference
Folic acid conjugated nanomedicines	Folic acid-cytotoxic drug conjugates	Folic acid-maytansinoid conjugates	Marked antiproliferative effect in ovarian, colon, nasopharyngeal carcinoma, lung, and cervical cancer (FR positive). No-activity in melanoma and breast cancer (FR negative)	Pre-clinical	[27,28]
		Folic acid-5-fluorouracil conjugates	Antitumor effect in colon cancer that overexpresses FRs	Pre-clinical	[30]
		Folic acid-methotrexate-arabinogalactan conjugates	Cytotoxic effect in leukemia models	Pre-clinical	[31]
		Folic acid-SB-T-1214 conjugates	Cytotoxic effect in blood, breast, and ovarian carcinomas overexpressing FRs.	Pre-clinical	[32]
		Folic acid-mitomicyn conjugates	Cytotoxic activity in lung adenocarcinoma models that overexpress FR	Pre-clinical	[33]
		Folic acid-bleomicyn conjugates	Cytotoxic effect in ovarian cancer (FR positive)	Pre-clinical	[34]
		Folic acid-camptothecin conjugates	Cytotoxic effect in FR positive cancer cells of the mouth	Pre-clinical	[37]
		Folic acid-polyethylene glycol-rhaponticin conjugates	Potent cytotoxic effect in FR positive epithelial and KB cancer cells. No-activity in FR-negative breast cancer cells	Pre-clinical	[38]
		Folic acid conjugated to a vinca alkaloid	Potent effect in ovarian cancer models (FR positive). This formulation is under clinical research	Clinical trial	[39]
		Folic acid conjugated to solubilizing peptide moiety (BMS-753493)	Phase I clinical studies in ovarian, colorectal, lung, and mammary tumors overexpressing FRs. An objetive anticancer response was not detected.	Clinical trial	[76]
		Folic acid conjugated to tubulysin (EC-1456)	Potent cytotoxic effect in vintafolide resistant FR-positive KB cancer cells. Phase I clinical studies in non-small lung cancer and ovarian cancer	Clinical trial	[77]
		Folic acid-Paclitaxel conjugates	No higher effect compared with unconjugated drugs in colon cancer cells that overexpress FR	Pre-clinical	[40]
		Folic acid-polyethylene glycol-cisplatin conjugates	No higher effect compared with unconjugated drugs in lung cancer cells that overexpress FR. However, a higher uptake was detected	Pre-clinical	[41]
	Folic acidfunctionalized nanoparticles	Folic acid-coated pH-sensitive liposomes loaded with doxorubicin	Cytotoxic effect in breast cancer models (FR positive)	Pre-clinical	[45]
		Folic acid-coated pH-sensitive liposomes loaded with paclitaxel		Pre-clinical	[46,47]
		Folic acid-coated non-pH-sensitive liposomes loaded with doxorubicin		Pre-clinical	[49]
		Folic acid-coated silica mesoporous nanoparticles loaded with topotecan	Cytotoxic effect in retina cancer models (FR positive)	Pre-clinical	[52]
		Folic acid-coated silica mesoporous nanoparticles loaded with cisplatin	Cytotoxic effect in cervical cancer models (FR positive)	Pre-clinical	[51]
		Folic acid-coated silica mesoporous nanoparticles loaded with doxorubicin		Pre-clinical	[50]
		Folic acid-coated Poly(lactic-co-glycolic acid) nanoparticles loaded with cisplatin		Pre-clinical	[53]
		Folic acid-Alendronate-coated Poly(lactic-co-glycolic acid) nanoparticles loaded with paclitaxel	Cytotoxic effect in non-small lung cancer overexpressing FR	Pre-clinical	[54]
		Folic acid-coated Poly(lactic-co-glycolic acid) nanoparticles loaded with paclitaxel and cisplatin	Non-coated and folic acid coated nanoparticles showed a similar tumor growth inhibition in FR-positive lung tumo models developed in mice	Pre-clinical	[55]
		Folic acid-functionalized iron oxide condensed colloidal magnetic clusters containing doxorubicin	Cytotoxic activity in FR-positive triple-negative breast cancer	Pre-clinical	[56]
		Folic acid-functionalized iron oxide nanoparticles	Cytotoxic activity in FR-positive cervical cancer	Pre-clinical	[57]
		Folic acid-coated liposomes loaded with BIM-S plasmid	Anticancer effect in non-small lung cancer. The formulations were designed for tumor associated macrophages	Pre-clinical	[61]
		Folic acid-functionalized-liposomes loaded with doxycycline		Pre-clinical	[63]
		Folic acid-coated chitosan nanoparticles loaded with signaling transducers and activators of transcription 3	Anticancer effect in Lewis lung adenocarcinoma. The formulations were designed for tumor associated macrophages	Pre-clinical	[64]
Anti-FR-monoclonal antibodies coupled nanoformulations	Antibody- drugconjugates	Farletuzumab conjugated with eribulin	Anticancer effect in non-small lung cancer overexpressing FR	Clinical trial	[68]
		Anti-FRα-monoclonal antibody-maytansinoid conjugate (mirvetuximab soravtansine)	Higher anticancer activity in ovarian and non-small lung carcinomas overexpressing FRα. Several clinical trials are ongoing to evaluate its efficacy as monotherapy or in combination with chemo and other immmunotherapeutic drugs in ovarian cancer patients	Clinical trial	[69,70,71,72,73,74]
	FR-targeted-nanoparticles	Antibody-conjugated gold-coated magnetite nanoparticles	Anticancer effect in triple-negative breast cancer overexpressing FR	Pre -clinical	[75]

## 3. Folic Acid-Targeted Nanoformulations in Inflammatory Diseases

**Figure 5:**
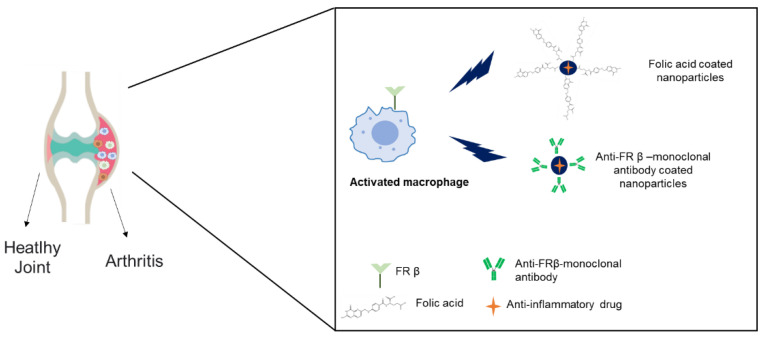
Summary of FR-targeted nanoformulations developed for inflammatory diseases.

Macrophages play a major role in the development and progression of chronic inflammatory diseases such as rheumatoid arthritis, osteoarthritis, inflammatory bowel diseases, and endometriosis, among others. As aforementioned, activated macrophages overexpress FRβ and the development of targeted nanoformulations represents a new opportunity in the treatment of these diseases [78,79] (Figure 5). Table 2 displays the nanoformulations that target activated macrophages via FR in inflammatory diseases. 

### 3.1. FR-Targeted Nanoformulations in Rheumatoid Arthritis

#### 3.1.1. Folic Acid-Coated Nanoparticles

In rheumatoid arthritis, a chronic autoimmune inflammatory disease, activated macrophages release inflammatory cytokines that trigger inflammation, cartilage damage, and bone destruction that result in pain, stiffness, swelling, and a functional impairment. Nowadays, some of the main treatment options are characterized by a low bioavailability at the action site, high clearance, and a limited selectivity, requiring the administration of high doses to obtain therapeutic efficacy [80]. The administration of high doses may result in a high systemic toxicity. In this way, the development of FR-targeted nanoformulations would allow a selective location of the drug at the activated macrophages, increasing the therapeutic efficacy and reducing the need for the administration of high doses and, consequently, preventing the high systemic toxicity. 

Methotrexate represents a main treatment option for rheumatoid arthritis. However, some patients are unresponsive, and the administration of high doses imply a potential risk of systemic toxicity. With the aim of improving its therapeutic efficacy, liposomes and polymeric-lipidic nanoparticles functionalized with folic acid and loaded with methotrexate have been developed. These nanoformulations have demonstrated to be efficiently internalized by activated macrophages and have been shown to increase the effect of methotrexate in a rodent model of arthritis [45,81,82]. Folic acid-coated double liposomes loaded with both methotrexate and prednisolone have also demonstrated to be effective in a rat model of arthritis, reporting a synergistic effect [83]. In fact, this represents another advantage for the use of these targeted nanocarriers, the localization of several drugs at the action site at the same time, potentiating the individual therapeutic effect of each agent. 

Dendrimers have also been conjugated to folic acid and loaded with methotrexate, showing to be effective for the selective delivery of this anti-inflammatory agent at the inflammation sites with a promising activity in a rat model of arthritis, reducing the ankle swelling, paw volume, cartilage damage, and bone resorption, parameters that are characteristics of an arthritis-induced inflammation [84,85]. 

Mesoporous silica-coated-gold nanoparticles functionalized with folic acid and loaded with methotrexate were effectively internalized by activated macrophages and showed the synergistic effect of gold nanoparticles (photothermal therapy) and methotrexate in the inhibition of arthritis progression in rats [86]. Albumin nanoparticles coated with folic acid and loaded with etoricoxib, also revealed remarkable targeting activity to activated macrophages in rheumatoid arthritis [87]. Finally, pH-sensitive polyethylene glycol-chitosan-based nanoparticles coated with folic acid and loaded with methotrexate were also effective to exert an anti-inflammatory effect in a rat model of rheumatoid arthritis as demonstrated by the reduction in arthritic signs and the levels of pro-inflammatory cytokines in treated mice [88]. 

**Table 2:** FR targeted nanoformulations designed for the treatment of inflammatory diseases.

Formulation		Disease	Stage	References
Folic acid coated nanoparticles	Folic acid-functionalized liposomes loaded with methotrexate	Rheumatoid arthritis	Pre-clinical	[81]
	Folic acid-functionalized lipid—polymeric nanoparticles loaded with methotrexate		Pre-clinical	[82]
	Folic acid-coated double liposomes loaded with both methrotrexate and prednisolone		Pre-clinical	[83]
	Folic acid-conjugated dendrimers loaded with methotrexate		Pre-clinical	[84]
	Folic acid-functionalized mesoporous silica-coated-gold nanoparticles loaded with methotrexate		Pre-clinical	[86]
	Folic acid-coated albumin nanoparticles loaded with etoricoxib		Pre-clinical	[87]
	Folic acid-coated pH-sensitive polyethylene glycol-chitosan-based nanoparticles loaded with methotrexate		Pre-clinical	[88]
Anti FRβ monoclonal antibody coated formulations	Anti-FRβ antibody-functionalized Cholesterol grafted chitosan nanoparticles loaded with methotrexate		Pre-clinical	[89]
Folic acid coated nanoparticles	Folic acid-coated poly(lactic-co-glycolic acid) nanoparticles loaded with resveratrol	Ulcerative Colitis	Pre-clinical	[90]

#### 3.1.2. Anti-FRβ Monoclonal Antibody-Coated Nanoformulations

The use of ligands specifically recognized by FRβ subtype could represent an advantage vs. the use of non-selective ligands like folic acid, as activated macrophages overexpress FRβ. In fact, Feng and co-workers developed a specific FRβ-specific human monoclonal antibody that was able to select FRβ-positive activated macrophages isolated from synovial fluid cells of rheumatoid arthritis patients [91]. This antibody represents a potential strategy to develop targeted drug conjugates or to functionalize nanocarriers for a more selective delivery of anti-inflammatory agents at the inflammation sites, as it specifically targets FRβ, which shows a more restricted distribution than FRα. 

Cholesterol-grafted chitosan nanoparticles functionalized with anti-FRβ antibody and loaded with methotrexate have been shown to exert an anti-inflammatory and anti-edema effect in a rat model of arthritis as demonstrated by the reduction in the paw volume and the serum cytokine expression [89]. 

### 3.2. FR-Targeted-Nanoformulations in Inflammatory Bowel Disorders

The term of inflammatory bowel disorders is mainly used to describe two diseases that imply an inflammation of the gut: Chron’s disease and ulcerative colitis. In these conditions, macrophages play a main role as they are activated and release pro-inflammatory cytokines such as TNF-α that produce the inflammation of the gut. Targeting activated macrophages may represent a good treatment strategy [92]. For example, folic acid-coated poly(lactic-co-glycolic acid) nanoparticles loaded with resveratrol showed colonic anti-inflammatory activity in rats, being useful for colitis treatment [90] and demonstrating the promising use of FR-targeted-nanoformulations to obtain selective delivery of the anti-inflammatory agents at the inflammation sites in this disorder. 

## 4. FR-Targeted Nanoparticles to Cross the Blood–Brain Barrier

FRα has also been found expressed in the plexus choroid, and FR-targeted formulations could be a good approach to overcome and cross central nervous system (CNS) barriers. In fact, Kuplennik and co-workers designed polymeric nanosystems functionalized with an FRα–folic acid complex and labelled with a fluorescent dye. Interestingly, this coating increased the passage of the nanoparticles through a choroid plexus epithelial cell monolayer. In vivo studies in mice, it was also demonstrated that modified nanoparticles were able to reach the brain [93], suggesting that this nanoformulation could be a good carrier to improve the access of drug to CNS. Despite these promising results, this formulation is very complex, especially considering its clinical translation. However, it indicates the potential utility of FR targeting to overcome access barriers to the CNS system. Venishetty and co-workers designed a simpler formulation consisting of folic acid grafted solid nanocapsules loaded with docetaxel and demonstrated that folic acid coating improved the delivery of docetaxel in the brain, as the FR-targeted formulation exhibited around a 44-fold higher brain permeation coefficient than conventional docetaxel. This study indicates that FR targeting could be a good strategy to deliver drugs in the brain [94].

## 5. FR-Targeted Formulations as Imaging Strategy 

FR targeting has also been used to evaluate FRs expression, to predict if the administration of FR-targeted therapies is a good treatment option and to monitor the treatments. In this way different FR-targeted imaging tools have been developed for cancer and inflammatory diseases such as rheumatoid arthritis and osteoarthritis. Table 3 displays the FR-targeted nanoformulations designed for imaging purposes. 

### 5.1. Cancer Disease

Several highly specific and sensitive methods are currently available to determine FRs expression in tumors such as immunohistochemistry, polymerase chain reaction, and in situ hybridization. However, their clinical use shows limitations as a biopsy is required, which is usually taken only once of a single lesion, providing sometimes incomplete information as the expression of FRs can be heterogeneous in cancer disease [95,96], especially when compared the primary lesion with distant metastases or during the treatment with the appearance of resistances. In this way, other techniques are required, and several radiolabeled imaging tools have been developed, as they provide a non-invasive method to evaluate FRs expression using positron emission tomography (PET) and single-photon emission computed tomography (SPECT) techniques [97]. This is the case of chimeric monoclonal antibody MOv18 IgG and F(ab′)2 fragments radiolabeled with ^125^I that have demonstrated the ability to identify tumor lesions of the ovary in patients [98]. [^111^In]In-diethylenetriaminepentaacetic acid (DTPA)-folic acid and etarfolatide ([^99m^Tc]Tc-EC20), a FR ligand radiolabeled with technetium-99, also demonstrated to be useful for the diagnosis of ovarian cancer [99] by SPECT, especially [^99m^Tc]Tc-EC20, which has been used to predict if FR-targeted chemotherapy would be useful in ovarian cancer patients [100,101]. 

PET radiotracers have also been developed with promising results for FR-positive tumor visualization. Conjugates such as [^18^F]F- folic acid derivative, 3′-Aza-2′-[^18^F]F-fluorofolic, and [^18^F]F-fluoro-deoxy-glucose folate were efficiently accumulated in KB-derived tumors (FR positive) developed in mice, with a limited accumulation in non-malignant tissues. This tumor accumulation was mediated by FR targeting, as it was avoided with the saturation of FRs by the previous administration of free folic acid [102,103,104]. [⁶⁸Ga]Ga-DOTA-folate was also accumulated in this tumor type, while it showed a low uptake in HT1080-derived tumors (FR negative) [105]. Similar results have been obtained with [⁶⁸Ga]Ga-NODAGA-folate, and [⁶⁸Ga]Ga -NOTA-folate, being also accumulated in KB-derived tumors. The latter even demonstrated a higher in vivo performance than ^99^mTc-EC20 [106,107]. ^68^Ga-labeled compounds have the disadvantage of a short half-life (around 68 min) and the advantage of a one-step radiolabeling. By contrast, ^18^F-tracers, which, although have a slightly higher half-life (110 min), require several radiolabeling steps. Farletuzumab, the specific anti-FRα- monoclonal antibody that exhibits a cytotoxic effect in ovarian cancer, has also been radiolabeled with indium-111 and iodine-131 for the use in radioimmunoscintigraphy and possibly radioimmunotherapy of FR-expressing carcinomas. These conjugates were even evaluated in patients with metastatic ovarian cancer, showing promising results as diagnostic tool [108].

Folate-radiolabeled conjugates that additionally contain an albumin-binding entity were more effective. Müller et al. showed that ^177^Lu-labeled-folate conjugates with this albumin binder (named as cm09) showed a higher tumor-to-background ratio (e.g., around 5–6-fold higher tumor-to-kidney ratio was achieved) compared to conjugates without albumin binder in KB tumor derived developed in mice. This was attributed to the increase in their blood circulation time due to their binding to plasmatic albumin [109]. Similar results were also obtained with [^18^F]F-fluorodeoxyglucose-folate and [^47^Sc]Sc-DOTA-folate containing an albumin binder, also showing a higher tumor-to-kidney ratio than conjugates without albumin binding properties [110,111]. Terbium-labelled albumin-binding and folate conjugates were also effective. [^152^Tb]Tb-folate and [^155^Tb]Tb-folate were effective probes for the visualization of KB-derived tumors by PET and SPECT, respectively, with a high tumor-to-background ratio [112]. Nevertheless, ^64^Cu-labeled and ^55^Co-labeled folate conjugates with albumin-binding capacity did not show a higher tumor-to kidney ratio, suggesting that the other radioconjugates could be a better option to the visualization of FR-positive tumors [113,114]. Finally, Guzik et.al demonstrated that [^177^Lu]Lu-N5, N10-dimethyltetrahydrofolic acid (MTHF) containing an albumin-binding entity was more effective than ^177^Lu-labeled albumin-binding folate conjugates, due to the selective targeting of MTHF to FRα, the FR that is overexpresses in tumors [115]. 

Interestingly, some FR-targeted radioconjugates have also demonstrated an anticancer activity, being useful as theranostic platforms. In the case of folate conjugates, [^177^Lu]Lu-cm09 demonstrated promising anticancer activity in KB-derived tumors. The administration of this conjugate at doses in the range of 7–20 MBq delayed the growth of KB tumors and increased the survival rate in mice. The best results were found at doses of 20 MBq, as the 80% of the tumors completely disappeared [109]. Similar results were reported with ^161^Tb-labeled albumin-binding folate conjugates that also produced a complete tumor remission in the 80% of the treated mice (KB-derived tumor was also used in this study as FR-positive tumor model). ^149^Tb-labeled albumin binding folate conjugates were also effective, but a lower response was detected with a 30% of complete remission observed [112]. [^47^Sc]Sc-DOTA-folate also demonstrated to reduce the growth of KB-derived tumors at doses of 10MBq, increasing the survival of mice by more than 50% [111]. A recent study carried out in IGROV-1 (ovarian cancer cell line overexpressing FR)-derived tumors in mice compared the efficacy of several folate radioconjugates ([^47^Sc]Sc -folate, [^177^Lu]Lu-folate and [^90^Y]Y-folate) showed a similar tumor growth inhibition when administered at doses of 12.5, 10, and 5 MBq, respectively, to obtain the same estimated absorbed tumor dose (~21 Gy) [116]. Finally, in NF9006-derived tumors (a breast cancer model) [^177^Lu]Lu-folate sensitized the tumors to anti-CTLA-4 immunotherapy, increasing the survival rate of the mice from around 19 days to more than 70 days. As monotherapy, this radiolabeled conjugate had a minor effect [117].

Non-radiolabeled strategies for FR imaging based on fluorescent conjugates have also been designed. Unlike radiolabeled agents, which must be used preoperatively, fluorescence agents could be used in combination with real-time imaging to improve the detection of lesions during surgery. For example, folic acid-fluorescein isothiocyanate conjugates, also known as EC17, have been developed and evaluated in patients with ovarian cancer with promising results [118]. 

Nanoparticles functionalized with folic acid or anti-FR-antibodies have also been designed and developed as nanoimaging tools in cancer disease. In this way, mesoporous silica nanoparticles conjugated with folic acid and loaded with a fluorescent dye demonstrated a selective uptake in pancreatic carcinoma models, being a good platform for tumor precision therapy [119]. Silica nanoparticles coated with folic acid and an entrapped phosphorescent compound have also been designed and evaluated in cervical cancer cell lines [120]. An antibody-functionalized nanoprobe that targets FRα has also been prepared for Raman imaging. This nanosystem, which consists of gold nanostars covered with a silica shell that was functionalized with anti-FRα-monoclonal antibodies, demonstrated to be useful as an imaging tool in ovarian cancer models developed in mice. Although these results are promising, the clinical translation of this system is currently limited by the lack of a commercial wide-field Raman-imaging camera system [121].

Nanotubes consist of other nanostructures that are garnering interest for drug delivery, and FR-targeted nanotubes have been designed. For example, Wang and co-workers developed Ni–folate biomolecule-based coordination complex nanotubes loaded with cisplatin. In HeLa cells (high FR expression) this formulation exhibited higher cytotoxic effect than free cisplatin. On the contrary, in HELF cells (low FR expression) the activity of free cisplatin was higher. This is attributed to the higher uptake of folic acid coated nanotubes in HeLa cells than in HELF cells (around 34% and 98% after 24 h of incubation) [122]. 

Finally, FR targeted nanoparticles with theranostic purposes have been designed. For example, mesoporous silica nanoparticles loaded with perfluorohexane and coated with indocyanine green (a photosensitizer agent used in photodynamic therapy) and folic acid have demonstrated to achieve tumor ultrasonic (US)/near-infrared fluorescence (NIRF) imaging in animal models and to inhibit cancer proliferation in FR-positive cells [123]. Polymeric nanoparticles containing paclitaxel, indocyanine green, and perfluorohexane, and coated with folic acid were also effective as a theranostic nanoplatform in animals [124]. Gold-albumin nanoparticles coupled with folic acid and loaded with doxorubicin were also effective. This nanoformulation exhibited a higher and selective anticancer efficacy in vitro. While in FR-positive gastric cancer cells (MGC-803) this nanoformulation showed a higher antiproliferative effect than free doxorubicin, in FR-negative gastric cancer cells (GES-1) it showed a lower effect than free doxorubicin. This higher anticancer effect in FR-positive tumors was also demonstrated in vivo using MGC-803-derived tumors in mice, showing a tumor growth inhibition of around 63% and 75% in free doxorubicin and targeted nanoparticles treated mice, respectively. Secondly, this formulation was useful to visualize the tumors using computed tomography imaging [125].

### 5.2. Inflammatory Diseases 

FR targeting has also been exploited for the identification and visualization of inflamed sites in several chronic inflammatory diseases. For example, folic acid-coated iron oxide nanoparticles have demonstrated to be an effective magnetic resonance imaging probe in a rat model of arthritis [126]. 

Radiolabeled liposomes coated with folic acid were useful for the visualization of the inflammation sites in a rodent model of atherosclerosis. Moreover, this formulation, loaded with betamethasone demonstrated to be effective for the imaging of the inflammation sites of a model of ulcerative colitis, simultaneously exerting a therapeutic effect [127].

**Table 3:** FR-targeted nanoformulations for imaging or theranostic purposes in cancer and inflammatory diseases: PET: Positron emission tomography, SPECT: Single-photon emission computed tomography, MRI: magnetic resonance imaging, NIFI: Near infrared fluorescence imaging.

	Formulation Type	Composition	Indication	Imaging Technique	Stage	Reference
Cancer	Radiolabeled FR ligands	Etarfolatide^®^ [^99m^Tc]Tc-EC20	Identification of tumors overexpressing FRs to predict when treatment with FR-targeted therapies is a good strategy This formulation is under clinical research.	PET	Clinical trial	[100,101]
		3′-aza-2′-[^18^F]F-fluorofolic acid	Visualization of FR-positive tumor masses, evaluated in vivo in KB-derived tumors developed in mice.	PET	Pre-clinical	[102]
		[^18^F]F-folic acid derivative		PET	Pre-clinical	[103]
		[^18^F]F-fluorodeoxyglucose-folate		PET	Pre-clinical	[104]
		[⁶⁸Ga]Ga-DOTA-folate	Visualization of FR-positive tumor masses, evaluated in vivo in KB-derived tumors developed in mice. No accumulation in HT1080-derived tumors (FR negative).	PET	Pre-clinical	[105]
		[⁶⁸Ga]Ga -NODAGA-folate	Visualization of FR-positive tumor masses, evaluated in vivo in KB-derived tumors developed in mice.	PET	Pre-clinical	[106]
		[⁶⁸Ga]Ga -NOTA-folate	Visualization of FR-positive tumor masses, evaluated in vivo in KB-derived tumors developed in mice. Better in vivo performance than ^99^mTc-EC20.	PET	Pre-clinical	[107]
		^177^Lu-labeled folate conjugates containing an albumin binder	Visualization of FR-positive tumor masses, evaluated in vivo in KB-derived tumors developed in mice. Better than conjugates without albumin binder. Anticancer activity in KB-derived tumors, ovarian cancer cell line overexpressing FR and in vivo breast cancer model.	PET	Pre-clinical	[108,116]
		[^18^F]F-fluorodeoxyglucose-folate with albumin-binding capacity	Visualization of FR-positive Kidney tumor masses. Better than conjugates without albumin binder.	PET	Pre-clinical	[109]
		^47^Sc-labeled folate conjugates containing an albumin binder	Visualization of FR-positive Kidney tumor masses. Better than conjugates without albumin binder. Anticancer activity in KB-derived tumors and ovarian cancer cell line overexpressing FR.	PET	Pre-clinical	[110]
		^152^Tb-labeled folate conjugates containing an albumin binder	Visualization of KB-derived tumors.	PET	Pre-clinical	[111]
		^155^Tb-labeled folate conjugates containing an albumin binder	Visualization of KB-derived tumors.	SPECT	Pre-clinical	[111]
		^64^Cu-labeled folate conjugates containing an albumin binder	visualization of FR-positive tumors. Lower tumor-to-kidney ratio compared with other radiolabelled conjugates.	PET	Pre-clinical	[112]
		^55^Co-labeled folate conjugates containing an albumin binder	Visualization of FR-positive tumors. Lower tumor-to-kidney ratio compared with other radiolabelled conjugates.	PET	Pre-clinical	[113]
		^177^Lu-labeled MTHF conjugates containing an albumin binder	Visualization of FR-positive tumor masses due to selective targeting to FRα.	PET	Pre-clinical	[114]
		[^161^Tb]Tb-albumin binding folate conjugate	Theracnostic purpose. Tumor visualization and anticancer activity in KB-derived tumors.	PET	Pre-clinical	[111]
		[^149^Tb]Tb-albumin binding folate conjugate	Theranostic purpose. Tumor visualization and anticancer activity in KB-derived tumors.	PET	Pre-clinical	[111]
		[^90^Y]Y-albumin binding folate conjugate	Theranostic purpose Anticancer activity in ovarian cancer cell line overexpressing FR.	PET	Pre-clinical	[115]
	Radiolabeled anti FR-monoclonal antibodies	[^111^In]In-farteluzumab(anti-FRα- monoclonal antibody) and [^131^I]I- farteluzumab	Radioimmunoscintigraphy and possibly radioimmunotherapy of ovarian carcinomas.	SPECT	Pre-clinical	[108]
	Fluorescent conjugates	Folic acid-fluorescein isothiocyanate conjugates (EC17)	Visualization of tumor masses in ovarian cancer patients during the cytoreductive surgery.	NIFI	Clinical trail	[118]
	FR targeted nanoparticles	Mesoporous Silica nanoparticles coated with folic acid and loaded with a fluorescent agent	Visualization of pancreatic tumors overexpressing FR.	NIFI	Pre-clinical	[119]
		Silica nanoparticles coated with folic acid and loaded with a phosphorescent agent	In vitro evaluation of FR expression in cervical cancer cells.	Phosphorescence Lifetime Imaging	Pre-clinical	[120]
		Gold nanostars covered with a silica shell that is functionalized with anti-FRα-monoclonal antibodies	Visualization of ovarian tumors	Raman imaging	Pre-clinical	[121]
		Mesoporous silica nanoparticles loaded with perfluorohexane and coated with indocyanine green and folic acid	Treatment and imaging or breast carcinomas	NIFI	Pre-clinical	[123]
		Folic acid-coated nanoparticles loaded with perfluorohexane and paclitaxel green indocyanine		Ultrasound imaging	Pre-clinical	[124]
		Gold-albumin nanoparticles coupled with folic acid and loaded with doxorubicin	Treatment and imaging or FR-positive gastric cancer cells	Tomography imaging	Pre-clinical	[125]
Arthritis	FR targeted nanoparticles for imaging	Folic acid-coated iron oxide nanoparticles	Inflammation site visualization	MRI	Pre-clinical	[126]
Ulcerative colitis	FR targeted nanoparticles for theragnosis	Liposomes coated with folic acid and loaded with betamethasone and a fluorescent dye	Inflammation site visualization and anti-inflammatory effect	NIFI	Pre-clinical	[127]

## 6. Advanced Folate Receptor Targeted Nanomedicines in Clinic

### 6.1. Etarfolide

As aforementioned, Etarfolide, which consists of [^99m^Tc]Tc-EC20, has been used for the identification of suitable cancer patients (those who overexpress FRs) for vintafolide administration [100,101]. Moreover, this radiotracer can be also useful for the localization of tumoral masses during the cytoreductive surgeries. This has been evaluated in ovarian cancer patients improving the surgeries. However, it should be considered that FRs are also expressed in kidneys, liver, and bone narrow, and although their expression is much lower compared with tumors, this tracer can be accumulated at these sites and make it difficult to identify tumors that are located in the vicinity of these organs [128]. Interestingly, a phase I study undertaken in healthy volunteers has reported that the previous administration of a small amount of folic acid with the aim of blocking these receptors located in heathy cells can resolve this limitation, as in cancer cells, the FRs expression is higher and tumor imaging would not be affected [129]. Other limitation of the use of Etarfolide is that FRs, specifically FRβ, are overexpressed in activated macrophages, internalizing this radiotracer. Consequently, the areas of infection and inflammation may falsely appear as tumors overexpressing FRs [130]. In this way, as tumors overexpress FRα the use of selective ligands to this subtype would resolve this problem. This is the case of [^99^Tc]Tc-MTHF [131] or the [^177^Lu]Lu-6R-5-MTHF containing albumin binder, which showed a high tumor-to-kidney ratio compared to ^177^Lu-labeled folic acid conjugates containing albumin binder [115]. 

Apart from cancer disease, etarfolide can also be useful for inflammatory diseases due to the overexpression of FRβ in activated macrophages. Preclinical studies have demonstrated the utility of this radiotracer to evaluate the participation of these receptors in inflammatory diseases such as arthritis [132,133]. In fact, a study in patients with rheumatoid arthritis has demonstrated the utility of this tracer to evaluate the degree of the disease, being even more effective than the physical examination [134]. 

Finally, a preclinical study has also demonstrated that etarfolide can detect both stable and vulnerable atherosclerotic plaques in a mouse model of atherosclerosis due to the participation of activated macrophages in this pathology [135]. 

### 6.2. Vintafolide

As aforementioned, this nanoformulation, which is also known as EC145 and Vynfinit^®^, consists of folic acid conjugated to a vinca alkaloid, specifically to desacetylvinblastine monohydrazide (DAVLBH), via a peptide linker. Due to the presence of folic acid, this conjugate enters the cell through FR-mediated endocytosis, disrupting the formation of the mitotic spindle that results in cell cycle arrest and, consequently, in cell death. This nanoformulation has been approved by FDA and EMA under the status of orphan drug for the treatment of ovarian cancer and non-small lung cancer. 

Leamon and co-workers demonstrated for the first time in 2006 that DAVLBH conjugated to folic acid via a peptide linker (this first conjugate was named EC140) showed a high affinity for FR-positive tumor cells (specifically for epidermal carcinoma of the mouth and lung) triggering a specific dose-response in vitro and in vivo. While EC140 conjugate showed a minor toxicity, the administration of unconjugated DAVLBH was much more toxic and less effective in M106- and KB-derived tumors. In contrast, 4-T1-derived breast tumors developed in mice (FR negative) both conjugated and unconjugated DAVLBH showed a similar toxicity and efficacy, indicating that FR targeting is a good strategy to increase the efficacy and reduce the toxicity of vinblastine in FR-positive tumors [136]. Further studies optimizing the linker and a new conjugate, named EC145, were obtained, which showed a higher efficacy and a lower toxicity compared with EC140 [137]. Due to these promising results, vintafolide conjugate has reached clinical investigation. 

Phase I studies undertaken in patients with refractory tumors, mainly gastrointestinal malignancies but also lung, ovarian, head and neck tumors, demonstrated that EC145 showed an acceptable safety profile when administered at doses of 2.5 mg on days 1, 3, 5, 15, 17, and 19 of a 28-day cycle, being nausea, vomiting and constipation the most common adverse effects [138,139], toxicities that were also found with unconjugated vinblastine [140]. A further study in patients with platinum-resistant ovarian cancer showed that the combination of vintafolide with liposomal doxorubicin was more effective than the single administration of liposomal doxorubicin, obtaining progression free survival rates of 5 and 2.7 months and overall survival rates of 18 and 12%, respectively, with both protocols. [101]. Other phase II study in ovarian cancer patients obtained similar results and demonstrated that the response to vintafolide correlates with FRs expression demonstrated by etarfolatide imaging, and indicating that the previous administration of this radiopharmaceutical is a useful tool to select the patients that are suitable for a FR-targeted-therapy [100]. Regarding the adverse effects, the combination of vintafolide and liposomal doxorubicin was in general well tolerated, showing similar toxicities than liposomal doxorubicin, unless leukopenia, neutropenia, abdominal pain, and peripheral sensory neuropathy that were statistically more frequent in the vintafolide plus doxorubicin treated patients [100]. In this case, the incidence of adverse effect was not related to FR levels [141]. Due to these results, a phase III clinical trial was launched in platinum resistant ovarian cancer patients that received vintafolide plus liposomal doxorubicin vs. liposomal doxorubicin plus placebo (NCT01170650). However, the study was discontinued as prespecified interim futility analysis revealed a lack of PFS in vintafolide treated patients [22]. 

Vintafolide has also been evaluated in patients with non-small cell lung cancer. In this study patients were treated with docetaxel or vintafolide as monotherapy or their combination. Vintafolide as monotherapy showed a lower efficacy than docetaxel with an ORR of 6% and 13% and a PFS of 1.6 and 3.3 months, respectively. However, their combination was more effective showing a significantly higher ORR or around 23% and a slightly higher PFS (4.2 months). A higher incidence of serious adverse effects (e.g., grade 3 and 4 neutropenia) were detected in the patients treated with both vintafolide plus docetaxel (72%) compared with docetaxel (55%) or vintafolide (10%) as monotherapy. [142]. 

Finally, the combination of vintafolide and other chemotherapeutics such as paclitaxel and carboplatin has also been evaluated in patients with advanced FR-positive endometrial carcinomas (NCT01688791). However, this study was terminated, and results have not been published. 

### 6.3. Mirvetuximab Soravtansine

This antibody drug conjugate consists of maytansinoid DM4, a maytansine derivative that shows a potent cytotoxicity, conjugated to mirvetuximab, a humanized monoclonal antibody targeting FRα. A phase I clinical study demonstrated that this compound, as monotherapy, was, in general, well-tolerated when administered once every three weeks at doses of 0.15–7 mg/kg, with fatigue, blurred vision, and diarrhea being the most common adverse effects. Some patients also experienced severe hypophosphatemia at doses of 5.0 mg/kg, and punctate keratitis at doses of 7.0 mg/kg, these symptoms being the main dose-limiting toxicities [71]. In patients with platinum-resistant epithelial ovarian cancer that overexpress FRα (this expression was evaluated by immunohistochemistry), this formulation, administered every 3 weeks at doses of 6.0 mg/k, exhibited an objective response rate of 26%, with 1 complete and 11 partial responses, and a median progression free survival of 4.8 months. Interestingly, these rates were higher in patients who had previously received three or fewer lines of therapy, with an objective response rate of 39% and a progression free survival of 6.7 months [72]. Administered at these doses, mirvetuximab soravtansine conjugate showed a higher efficacy in platinum-resistant ovarian cancer patients compared with conventional chemotherapy (paclitaxel, topotecan, or pegylated liposomal doxorubicin) with an objective response rate of 24 vs. 10% and a progression free survival of 4.8 vs. 3.3 months, being well tolerated. Consequently, it demonstrated a favorable benefit–risk profile [73]. In this resistant tumor, this conjugate was also effective in combination with bevacizumab, with no major toxicities, with diarrhea, blurred vision, nausea, and fatigue being the most common adverse effects. An encouraging efficacy with an objective response rate of 56% was observed in patients with a mild or high expression of FRα [74]. 

### 6.4. EC17 Conjugate

EC17 is an imaging probe that consists of folic acid conjugated to fluorescein isothiocyanate, which allows the detection of tumoral masses as it trends to accumulate in tumors via FR-mediated endocytoses. A study undertaken in patients with epithelial ovarian carcinoma demonstrated that this conjugate, injected during the surgery at doses of 0.3 mg/kg over a period of 10 min, allowed the visualization of the tumor areas without affecting the surgical procedure. Moreover, no serious toxicities were reported during or after the surgery that could be related to EC17 administration [118]. Another study obtained similar results when administered even at lower doses of 0.1 mg/kg, being able to identify both breast and ovarian tumor lesions. In fact, it was reported that this technique was able to detect ovarian tumor lesions that were not identified with palpation or inspection [143]. 

### 6.5. BMS-753493 Conjugate

BMS-753493 is a FR-targeted conjugate formed by the epothilone moiety, it consists of a macrolactone and an aziridine ring, conjugated to folic acid bearing solubilizing peptide moiety using a cleavable carbonate linker [144]. An in vivo study carried out in mice with tritium ^3^H labeled BMS-753493 conjugate demonstrated a significantly higher accumulation in M109-derived tumors overexpressing FR than in M109-derived tumors that lacks of these receptors, indicating an FR-mediated accumulation [145]. In fact, this formulation has been evaluated in humans with different solid tumors, mainly ovarian, colorectal, lung, and mammary tumors. A phase I clinical study demonstrated a maximum tolerated dose of 26 mg when administered at doses of 5 mg on day 1, 4, 8, and 11 every three weeks, and of 15 mg when used at doses of 2.5 mg on day 1, 2, 3, and 4 every three weeks. In general, this conjugate was well tolerated, with fatigue, transaminitis, gastrointestinal disorders, and mucositis being the dose-limiting toxicities. However, no objective responses were detected, and further clinical research was discontinued [76]. 

### 6.6. EC1456 Conjugate 

This formulation consists of tubulysin conjugated to folic acid via a reducible linker [146]. In vitro, in KB cells (FR positive) EC1456 was around 1000-fold more effective in absence of folic acid than when it was incorporated as benign competitor, indicating a FR selective activity. It also decreased the growth of KB-derived tumors in mice. Interestingly, it increases the effect of conventional antineoplastics when combined, especially with cisplatin, carboplatin, docetaxel, or bevacizumab, achieving 100% tumor cure in all these combinations. In KB-145–55 derived tumors (they are resistant to vintafolide) EC1456 was effective. While around 10% of tumor reduction was achieved in vintafolide treated mice, around 80% of tumor growth inhibition was detected with EC1456. All these results suggest that EC1456 conjugate could be a promising strategy to treat FR-positive tumors, even the vintafolide resistant ones [147]. 

Nowadays, several phase I clinical studies have been launched to evaluate its efficacy in patients with non-small cell lung cancer (NCT01999738) and ovarian cancer (NCT03011320). Preliminary results in lung cancer patients showed that EC1456 was in general well tolerated, recommending doses of 6.0 mg/m^2^ twice weekly or 12.5 mg/m^2^ every week. Gastrointestinal disorders, fatigue, metabolic changes, alopecia, and headache were common adverse effects [77]. 

## 7. Current and Future Perspectives 

In normal tissues, the expression of folate receptors is low and limited to cells that are important for embryonic development, like plexus choroid and placenta, and for folate reabsorption, being also expressed in the kidneys. However, in several pathological conditions, folate receptors are overexpressed in certain cells. This is the case of cancer cells and activated macrophages. This overexpression makes them a potential therapeutic target, in the treatment of cancer and inflammatory diseases, to obtain a selective delivery of drugs at altered cells level, and thus to improve the therapeutic efficacy and decrease the systemic toxicity of the pharmacological treatments. In this way, numerous folate receptor-targeted formulations have been designed and developed to improve cancer chemotherapy, mainly in lung, colon, and ovarian cancer, tumors that highly overexpress FRα, or anti-inflammatory therapies, mainly in rheumatoid arthritis. 

Two strategies have been used to achieve this targeting to folate receptors: the use of folic acid or other ligands with a high affinity for FRs, and the use of anti-FRs-monoclonal antibodies. In both cases, they are conjugated to the therapeutic agent or bound to the surface of a nanocarrier that is loaded with the drug. Currently, conjugation with folic acid is the most used strategy, probably due to its lower cost and greater ease of manipulation than monoclonal antibodies. Folic acid has a high affinity for folate receptors, and folic acid-conjugated compounds trend to accumulate at tumor sites and activated macrophages in cancer and inflammatory diseases. Binding of folic acid to folate receptors is mainly through the pterine ring (Figure 2). Folic acid pteroate moiety is buried inside the receptor pocket. However, the glutamic acid moiety of folic acid is exposed and sticks out the receptor entrance [15]. This explains why the conjugation of folic acid to drugs or nanoparticle surface is through this glutamate entity, usually through a linker such as polyethylene glycol. Numerous folic acid-conjugated nanoformulations have been designed and developed, including folic acid-cytotoxic drugs conjugates for the improvement of cancer chemotherapy, and nanoparticles functionalized with folic acid and loaded with antineoplastic or anti-inflammatory agents, for the treatment of cancer or inflammatory disorders. Although to a lesser degree than folic acid-based systems, nanoformulations containing anti-FR-monoclonal antibodies have also been studied, especially in cancer disease. In fact, this strategy has the advantage of allowing a FRα or FRβ selective targeting, which could be interesting, especially in the case of inflammatory disorders. Between both subtypes, FRα is more common, and it is the isoform that is usually expressed in normal tissues. On the contrary, FRβ is mainly limited to activated macrophages, and therefore targeting to FRβ would allow a more selective distribution at the inflammation sites or at the macrophages that are at the tumor microenvironment. 

Apart from therapeutic purposes, FR targeting also represents a good approach for imaging purposes. In cancer disease, this approach is useful to evaluate FRs expression in cancer cells, predict if the administration of FR-targeted therapies is a good treatment option, monitor the efficacy of the treatments or localize tumoral masses during the surgery. In inflammatory disorders, it could be useful to localize inflammation sites. Several FR-targeted imaging nanotools have been designed, especially for cancer imaging but also for inflammation localization, most of them based on radiolabeling and are detected using both PET and SPECT imaging techniques, or other based on the incorporation of a fluorescent entity like green indocyanine or fluorescein that are detected by near infrared fluorescence imaging. In fact, some formulations have reached the clinic, being used for the detection of tumoral masses during surgery and for the identification of patients with tumors that are suitable for FR-targeted chemotherapy. 

Currently, among all the applications of FR targeting, the one with the greatest potential is tumor treatment and imaging, especially of ovarian cancer, as FRα is overexpressed in the 90% of epithelial ovarian carcinomas, which represent more than 95% of ovarian malignancies and are the most common cause of gynecological cancer death. In fact, four formulations that have reached the clinic are mainly used in ovarian cancer patients for imaging during the cytoreductive surgery or to predict the potential use of FR-targeted chemotherapy. Interestingly, in all four cases they are conjugates of a targeting moiety (FR ligands or anti-FR monoclonal antibodies) and a cytotoxic drug or an imaging agent, probably due to the difficulties of the clinical translation of nanocarriers formulations. In addition to ovarian cancer, folate receptor targeting could be also useful in other tumors that also show a high overexpression of FR such as colon, endometrial, and non-small lung carcinomas.

## 8. Conclusions

It can be concluded that FR targeting represents a great strategy for precision medicine in cancer and inflammatory diseases, resulting in a useful tool for both diagnosis and treatment. In the case of cancer, currently, FR-targeted formulations are especially useful for ovarian cancer, as almost all the clinical studies have been developed in patients with this neoplasm. However, their utility could be also moved to other cancer types, such as endometrial carcinoma, with a couple of recently initiated clinical trials, and colon and lung carcinomas. FR targeting also represents a good approach to improve arthritis rheumatoid therapy, especially to improve the effect, and reduce the toxicity, of methotrexate, its most studied drug. 

## Data Availability

Not applicable.
